# Comparison of Clinical Outcomes of Persons Living With HIV by Enrollment Status in Washington, DC: Evaluation of a Large Longitudinal HIV Cohort Study

**DOI:** 10.2196/16061

**Published:** 2020-04-15

**Authors:** Jenevieve Opoku, Rupali K Doshi, Amanda D Castel, Ian Sorensen, Michael Horberg, Adam Allston, Michael Kharfen, Alan E Greenberg

**Affiliations:** 1 HIV/AIDS, Hepatitis, STD, and TB Administration DC Health Washington, DC United States; 2 Department of Epidemiology, Milken Institute School of Public Health George Washington University Washington, DC United States; 3 Kaiser Permanente Medical Group Rockville, MD United States

**Keywords:** HIV, DC Cohort, cohort studies, HIV clinical outcomes

## Abstract

**Background:**

HIV cohort studies have been used to assess health outcomes and inform the care and treatment of people living with HIV disease. However, there may be similarities and differences between cohort participants and the general population from which they are drawn.

**Objective:**

The objective of this analysis was to compare people living with HIV who have and have not been enrolled in the DC Cohort study and assess whether participants are a representative citywide sample of people living with HIV in the District of Columbia (DC).

**Methods:**

Data from the DC Health (DCDOH) HIV surveillance system and the DC Cohort study were matched to identify people living with HIV who were DC residents and had consented for the study by the end of 2016. Analysis was performed to identify differences between DC Cohort and noncohort participants by demographics and comorbid conditions. HIV disease stage, receipt of care, and viral suppression were evaluated. Adjusted logistic regression assessed correlates of health outcomes between the two groups.

**Results:**

There were 12,964 known people living with HIV in DC at the end of 2016, of which 40.1% were DC Cohort participants. Compared with nonparticipants, participants were less likely to be male (68.0% vs 74.9%, *P*<.001) but more likely to be black (82.3% vs 69.5%, *P*<.001) and have a heterosexual contact HIV transmission risk (30.3% vs 25.9%, *P*<.001). DC Cohort participants were also more likely to have ever been diagnosed with stage 3 HIV disease (59.6% vs 47.0%, *P*<.001), have a CD4 <200 cells/µL in 2017 (6.2% vs 4.6%, *P*<.001), be retained in any HIV care in 2017 (72.9% vs 59.4%, *P*<.001), and be virally suppressed in 2017. After adjusting for demographics, DC Cohort participants were significantly more likely to have received care in 2017 (adjusted odds ratio 1.8, 95% CI 1.70-2.00) and to have ever been virally suppressed (adjusted odds ratio 1.3, 95% CI 1.20-1.40).

**Conclusions:**

These data have important implications when assessing the representativeness of patients enrolled in clinic-based cohorts compared with the DC-area general HIV population. As participants continue to enroll in the DC Cohort study, ongoing assessment of representativeness will be required.

## Introduction

Cohort studies have commonly been used to examine the progression of a disease or an intervention and have also been shown to be an important tool in assessing health outcomes and effective treatments among study populations [1]. However, people who are approached and agree to participate in research studies may not completely represent the general population, and study results cannot necessarily be generalized [1-3]. Care providers may be biased as to which patients they approach to be in research studies, which may then lead to insufficient or unrepresentative recruitment [4-7]. Further, subjects may self-select for inclusion into the study based on perceived benefits of participation or incentives provided or may decline participation because of perceived obstacles to participation [7]. Perceived obstacles, including HIV stigma, access to care, economic challenges, and wariness to partake in research may hinder potential participants from engaging in research studies. These differences in perception may correlate with underlying demographics and outcomes, which result in selection bias in the data [7]. This self-selection, better known as participation bias, is a common occurrence in research studies and impacts the reliability of results [3,7].

It is, however, possible to evaluate differences and similarities between a cohort and the source population in a few different ways [8-15]. For instance, a study comparing data from the Ontario HIV Treatment Network with hospital records was able to classify individuals who participated in the study, those who declined to participate in the study, and those who were not approached at all [13]. Study participants tended to be older, white, men who have sex with men (MSM), and on antiretroviral therapy (ART) and have a longer duration of HIV infection [13]. Furthermore, while individuals who declined to participate had similar rates of viral suppression, individuals who were not approached tended to have higher rates of not being virally suppressed [13]. In another cohort study comprising MSM (HIV infected or not), it was found that compared with participants and those who later dropped out of the study, nonparticipants tended to have lower incomes and education levels and were less likely to identify as gay or bisexual and more likely to be nonwhite and married [8]. Interestingly, those who were lost to follow-up were most likely to be HIV positive [8].

The power and usefulness of findings from cohort studies rely upon the assumption that research participants represent the population from which they were drawn and, therefore, these findings can be generalized toward the total population. Biases stemming from differential patterns of enrollment may lead to overestimates or underestimates of the effectiveness of an intervention or outcome measure, particularly among nonparticipants [3,4]. Similarly, studies that provide prevalence estimates of specific risk factors or health-related outcomes can be affected by these biases, leading to over or underestimation of important population parameters [4].

Since 2011, the DC Cohort study has enrolled people living with HIV who receive care at one or more of 15 medical care sites in Washington, DC [16]. One objective of the study is to enroll a representative sample of people living with HIV disease in DC. The purpose of this analysis was to compare DC Cohort study participants to the general population of people living with HIV in DC who were not enrolled in the study and assess whether cohort participants are representative of people living with HIV in DC. This analysis sought to assist in determining whether demographic and clinical outcomes among cohort participants can be generalized to those diagnosed with HIV and living in DC.

## Methods

### Surveillance Data

HIV surveillance data from DC Health (DCDOH) enhanced HIV/AIDS Reporting System, the hepatitis surveillance registry, and the DC Public Health Information System were extracted. People living with HIV who were in DC at the end of 2016 were included in this analysis. People living with HIV in DC at the end of 2016 were defined as people (1) diagnosed with HIV; (2) whose last reported HIV lab result (eg, CD4 or HIV RNA) included a DC address and was reported between January 1, 2011, and December 31, 2016, to the DCDOH HIV/AIDS, Hepatitis, STD and TB Administration (HAHSTA); and (3) who were alive at the end of 2017. All lab-confirmed gonorrhea; chlamydia; primary, secondary, and early latent syphilis; and chronic hepatitis B virus (HBV) and hepatitis C virus (HCV) diagnoses were included. This work has been approved by both the DC Health and George Washington University School of Public Health institutional review boards.

### DC Cohort Study

The DC Cohort study is a prospective, longitudinal, observational cohort study whose primary goal is to contribute to improving the quality of care and treatment of HIV-infected patients in DC. Details of the design of the study have been previously described [17,18]. Briefly, children, adolescents, and adults diagnosed with HIV disease who receive medical care from at least one of 15 HIV care sites provide informed consent to participate in the study and have their data from electronic medical records (EMRs) extracted on a monthly basis. Sites for the DC Cohort study were methodically selected to include a variety of sites with respect to size, patient population served (by risk, race/ethnicity, age), and services provided [17]. The facilities included in the DC Cohort study represent the major HIV care sites in DC, including hospitals and hospital-based and community-based HIV clinics, with the exception of private providers and one DC-based hospital [17,19]. These HIV care sites are located in the 6 wards in DC where HIV prevalence is highest (out of 8 total wards). Of the 15 sites currently participating in the DC Cohort study, only 14 sites contributed to this analysis as one HIV care site began enrollment after 2016. All participants who consented to participate between January 1, 2011, and December 31, 2016, were included in this analysis [16]. Participants who lived outside of DC were excluded from this analysis as their data would not be routinely captured in the DCDOH HIV surveillance database.

### Data Match

DC Cohort study and DCDOH HAHSTA surveillance data are matched every 6 months as part of the study protocol. DC Cohort study data were matched based on an 11-key algorithm linking first name, last name, date of birth, and social security number [18-21]. Linkage keys range from including social security number or full first name, last name, and date birth to only including the first 3 letters of the first name, last name, and the date of birth year. Matches made through keys 7-11, which all consist of only partial first and last names and dates of birth, were manually reviewed and checked for accuracy (Table 1) [21]. Of the initial 275 patients who matched through keys 7-11, after deduplication nearly 30% (79/275) were not true matches and were eliminated from the dataset. Total matches were then validated using LinkPlus, a record linkage application. All linkages with a score of at least 80 were included in the final dataset. Data were then stratified by demographics, including current gender identity, race/ethnicity, median age, mode of HIV transmission, ever diagnosed with a sexually transmitted infection (STI), and ever diagnosed with confirmed chronic HBV or HCV. While surveillance data collects longitudinal data on STIs, HBV, and HCV, we limited diagnoses to those reported between 2011 and 2016 to mirror the enrollment period of the DC Cohort study patients. Although the DC Cohort study collects data from EMRs, for the purposes of this analysis DC Cohort study identification (ID) numbers were used to identify cohort participants, and only data from the DCDOH surveillance databases, and not the EMRs, were used to compare the two groups.

**Table 1:** Surveillance data matching algorithm.

Match level	Match criteria
Match 1	If social security number
Match 2	Else if, first name (first 6 letters), last name, date of birth
Match 3	Else if, last name (first letter), last name (letters 3 through 8), first name (letters 2 through 8), date of birth
Match 4	Else if, last name (first letter), last name (letters 3 through 8), first name (letters 2 through 8), birth month, birth year
Match 5	Else if, last name (first letter), last name (letters 3 through 8), first name (letters 2 through 8), birth day, birth year
Match 6	Else if, last name, first name (letters 1 through 2), date of birth
Match 7	Else if, last name (letters 1 through 3), first name (letters 1 through 3), date of birth
Match 8	Else if, last name (letters 1 through 4), first name (letters 1 through 4), birth year
Match 9	Else if, first name (letters 1 through 3), last name (letters 1 through 3), birth month, birth year
Match 10	Else if, first name (letters 1 through 3), last name (letters 1 through 3), birth day, birth year
Match 11	First name (letters 1 through 3), last name (letters 1 through 3), birth month, birth year

### HIV Disease Stage in 2017

Current US Centers for Disease Control and Prevention guidelines provide a classification system for assessing the severity of HIV disease based on CD4 cell counts and the presence of specific HIV-related conditions [22,23]. Stage 1 HIV disease is defined by having a CD4 count of more than 500 cells/µL or a CD4 percentage of more than 29%. Stage 2 is defined by having a CD4 count between 200 and 500 cells/µL or a CD4 percentage between 14% and 28%. Stage 3 (AIDS) infection is defined as having a CD4 count of less than 200 cells/µL, a CD4 percentage of less than 14%, or a diagnosed AIDS-related condition (ie, an HIV-related opportunistic infection). These stages of HIV disease were categorized using laboratory values from the last lab result reported on or before December 31, 2017.

### Receipt of HIV Medical Care

To measure receipt of HIV care, lab results reported to the DCDOH were further evaluated. Cases were considered to have received care if they had at least one lab result (CD4 or viral load [VL]) between January 1, 2017, and December 31, 2017 [22,23]. People living with HIV that did not show any evidence of having a lab result reported in 2017 were categorized as not engaged in care in 2017.

### Viral Suppression

“Ever virally suppressed” was defined as having at least one VL test result less than or equal to 200 copies/mL between 2011 and 2017. Viral suppression in 2017 was described as having a VL test result less than or equal to 200 copies/mL between January 1, 2017, and December 31, 2017 [22,23]. Time to viral suppression was defined as the length of time from first reported detectable VL to first reported VL lab result of less than or equal to 200 copies/mL among those who have ever been virally suppressed.

### Confirmed Chronic Hepatitis B Virus and Hepatitis C Virus Diagnoses

All positive hepatitis antibody tests are reported to DCDOH by all commercial laboratories conducting this testing in DC. All HBV and HCV labs reported to DCDOH and identified as chronic or probable were assessed. Chronic HBV and HCV lab results with a positive RNA test result were considered confirmed and included in this analysis.

### Statistical Analysis

Statistical analyses were performed using SAS version 9.4 (SAS Institute Inc). Univariate analysis using Pearson chi-square tests and *P* values for categorical data and analysis of variance for continuous data was performed to identify differences in cohort and noncohort participants with respect to demographics, comorbid conditions (ie, STIs, hepatitis), clinical and virologic outcomes (ie, CD4, VL, viral suppression), and receipt of HIV care. Multivariate log binomial regression was used to assess differences in clinical outcomes between DC Cohort and noncohort participants adjusting for demographics, time since HIV diagnosis, and mode of transmission.

## Results

At the end of 2016, there were 12,964 people living with HIV in DC, of which 5193 (40.1%) were DC Cohort study participants. Compared with nonparticipants, analysis showed that cohort participants were less likely to be male but more likely to be non-Hispanic black and have heterosexual contact as their HIV transmission risk (Table 2). Cohort participants had been living longer with HIV (12.6 years vs 10.7 years, *P*=.048) and were more likely to have a chronic HCV diagnosis but less likely to have been diagnosed with an STI between 2011 and 2016. There were no differences in median age at the end of 2017 or in chronic HBV diagnoses between the two groups.

**Table 2:** Demographic characteristics of DC Cohort and non-DC cohort participants living in DC as of December 2017 (n=12,964).

Characteristic		DC^a^ cohort n=5193 n (%)		non-DC cohort^b^ n=7771 n (%)	*P* value	
**Gender identity**					**<.001**	
	Male		3533 (68.0)	5818 (74.9)		
	Female		1580 (30.4)	1816 (23.4)		
	Transgender		80 (1.5)	137 (1.5)		
**Race/ethnicity**					**<.001**	
	White		515 (9.2)	1561 (20.1)		
	Black		4271 (82.3)	5399 (69.5)		
	Hispanic		302 (5.8)	582 (7.5)		
	Other^c^		105 (2.0)	229 (3.0)		
**Transmission risk**					**<.001**	
	MSM^d^		1977 (38.1)	3764 (48.5)		
	IDU^e^		768 (14.8)	604 (7.8)		
	MSM/IDU		198 (3.8)	219 (2.8)		
	Heterosexual contact		1571 (30.3)	2014 (25.9)		
	Perinatal		94 (1.8)	43 (0.6)		
	Other^f^		3 (0.1)	7 (0.1)		
	Not identified		582 (11.2)	1121 (14.4)		
Age in years^g^, median (IQR^h^)		50 (18)		48 (20)	.65	
Time since HIV diagnosis in years, mean (SD)		12.6 (6.9)		10.7 (7.4)	.048	
STI^i^ diagnosis between 2011-2016		906 (17.4)		1471(18.9)	.03	
Hepatitis B coinfection between 2011-2016		87 (1.7)		100 (1.3)	.07	
Hepatitis C coinfection between 2011-2016		282 (5.4)		345 (4.4)	.009	

^a^District of Columbia.

^b^Non-DC cohort participants include persons who have consented and subsequently withdrawn from the study and persons diagnosed with HIV and reported to the DC Health who were alive as of the end of December 2017.

^c^Other race includes mixed race individuals, Asians, Alaska Natives, American Indians, Native Hawaiian, Pacific Islanders, and unknown race.

^d^MSM: men who have sex with men.

^e^IDU: injection drug user.

^f^Other mode of transmission includes perinatal transmission, hemophilia, blood transfusion, and occupational exposure (health care workers).

^g^Age as of December 31, 2017.

^h^IQR: interquartile range.

^i^STI: sexually transmitted infection.

In evaluating clinical outcomes, DC Cohort study participants were more likely to have been diagnosed with stage 3 HIV disease, have a CD4 count of <200 cells/µL in 2017, and have received any HIV care in 2017 (Table 3). DC Cohort study participants were also more likely to have ever been virally suppressed and more likely to be virally suppressed in 2017 but were less likely to be suppressed within 2 years of HIV diagnosis. There was no difference in median CD4 count at the end of 2017 between the two groups.

After adjusting for gender identity, current age, race/ethnicity, time since HIV diagnosis, and mode of HIV transmission, DC Cohort study participants were 24% more likely to have received any care in 2017 (adjusted odds ratio 1.24, 95% CI 1.21-1.28), and over 10% more likely to ever have been virally suppressed (adjusted odds ratio 1.11, 95% CI 1.07-1.15; Table 4).

**Table 3:** Clinical characteristics of DC Cohort and non-DC cohort participants living in DC as of December 2017 (n=12,964).

Characteristic		DC^a^ cohort	Non-DC cohort^b^	*P* value
Ever stage 3 diagnosis (eg, AIDS, CD4 <200 cells/µL, or OI^c^), n (%)		3093 (59.6)	3652 (47.0)	<.001
Engaged in HIV care in 2017, n (%)		4336 (83.5)	5572 (71.7)	<.001
CD4 count (cells/µL) in 2017, median (IQR^d^)		618 (440)	610 (431)	.83
**CD4 count (cells/µL), most recent**				**.19**
	<200, n (%)	365 (8.5)	455 (8.4)	—
	200-500, n (%)	1159 (27.0)	1495 (27.6)	—
	>500, n (%)	2764 (64.5)	3473 (64.0)	—
Virally suppressed^e^ between 2011-2017, n (%)		4348 (83.7)	6070 (78.1)	<.001
Virally suppressed^e^ at last lab in 2017, n (%)		3189 (61.4)	3921 (50.5)	<.001
**Time to first known viral suppression^e^, n (%)**				**<.001**
	0-24 months	1472 (33.4)	2382 (39.2)	—
	>24 months	2876 (65.6)	3688 (60.7)	—

^a^DC: District of Columbia.

^b^Non-DC Cohort participants include persons who have consented and subsequently withdrawn from the study, as well as persons diagnosed with HIV and reported to the DC Health who were alive as of the end of December 2017.

^c^OI: opportunistic infection.

^d^IQR: interquartile range.

^e^Viral suppression defined as HIV RNA <200 copies/mL.

**Table 4:** Adjusted prevalence ratios for clinical characteristics of DC Cohort and non-DC cohort participants living in DC as of December 2017.

Factor^a^	APR^b^ (95% CI)
Model 1: retained in any care	1.24 (1.21-1.28)
Model 2: ever virally suppressed	1.11 (1.07-1.15)
Model 3: virally suppressed at last lab result in 2017	1.03 (0.97-1.02)
Model 4 (among those ever virally suppressed): suppressed ≥24 months versus 0-12 months	1.02 (1.08-1.14)

^a^Adjusting for gender identity; age on December 31, 2017; race/ethnicity; time since HIV diagnosis; and mode of HIV transmission.

^b^APR: adjusted prevalence ratio.

## Discussion

### Principal Findings

We sought to determine if the characteristics of a study cohort of consenting people living with HIV receiving care in DC were representative of the population of people living with HIV in a large urban city. When comparing DC Cohort study enrollees to that of the overall population of people living with HIV in DC, we identified notable demographic, disease transmission, and clinical differences. The greatest absolute differences with respect to demographics and disease transmission were observed in the proportion of those who identified as black, identified as female, or had a mode of HIV transmission of MSM, IDU, or heterosexual contact. While differences in race/ethnicity and gender identity were not expected, cohort data on people who refuse to participate in the DC Cohort study have identified differences in consenting with respect to sex at birth and race (data unpublished). Additionally, given the large sample size in our analysis, we may have been able to detect statistically smaller differences between the two groups [24-26]. Third, differences in race/ethnicity and mode of HIV transmission between DC Cohort and noncohort participants may be related to the clinics and care facilities to which cohort patients are enrolled.

Differential representation in the DC Cohort study may be explained in part by the characteristics of the participating clinic sites and demographics of the patients to whom they provide care. For example, although the largest HIV care providers and those that care for particular subpopulations are currently participating in the DC Cohort study, smaller and private health care facilities that may provide HIV care services to specific HIV-positive subpopulations such as those that have more non-Hispanic white or predominantly MSM populations are not currently included as recruitment sites. In the HIV Prevention Trials Network (HPTN) 065 study, also known as the Test, Link-to-Care Plus Treat (TLC-Plus) study, research was conducted in 6 major US cities using health centers, major hospitals, community-based organizations, and private medical facilities to enroll patients to assess the feasibility of expanding HIV testing across medical settings and providing incentives for improved health outcomes [27]. Of the care sites participating in HPTN 065, private medical practices accounted for 26.3% of all HIV care sites that participated in the DC arm of the study [27] and 40.1% of all non-Hispanic white MSM participants in the study (data unpublished), demonstrating that private medical practices provide HIV care to a substantial number of distinct populations, including white MSM. Laboratory and case report data reported to DCDOH have also shown that among newly diagnosed persons in 2017, private medical practices accounted for 32.6% of non-Hispanic white diagnoses and 46.4% of diagnoses among white MSM (data unpublished). As the DC Cohort study expands and continues to enroll clinical sites contributing data collection, these disparities may be reduced. Despite the differences demographically, length of time since HIV diagnosis and diagnoses of STIs, HBV, and HCV were similar across the two groups suggesting that analysis of these outcomes in the DC Cohort study population are likely to be fairly generalizable to people living with HIV in DC.

The evaluation of clinical outcomes revealed expected differences between DC Cohort and noncohort participants. Ever having stage 3 HIV disease (AIDS) was higher among DC Cohort participants. The DC Cohort appears to be consenting individuals who were diagnosed at a more advanced stage of HIV disease; individuals with declining health outcomes may be more likely to seek treatment and therefore have more opportunities to be approached for study enrollment. Having a history of AIDS may predispose an individual to opportunistic infections and non-AIDS conditions that are affected by prolonged viremia and immune activation such as cardiovascular events and certain cancers. Thus, studies in the DC Cohort that measure outcomes that are affected by ever having AIDS should be cautious about extrapolation of findings.

Receipt of any care in 2017 was 12% higher and viral suppression in 2017 was 11% higher among DC Cohort participants versus noncohort people living with HIV. These two key indicators of engagement in HIV care suggest a few possibilities: (1) since the DC Cohort study enrolls people at their site of care, it is likely that DC Cohort participants are more likely to be engaged in HIV care, (2) DC Cohort participants are more engaged in HIV care or the clinic is more active in engaging their patients in care, and/or (3) the proportion of noncohort people living with HIV who are no longer living in DC outweighs that of the cohort people living with HIV and the denominator used for this group in this analysis may be too large. Any of these explanations is possible. In an analysis evaluating a local HIV lab data exchange, DC residents diagnosed with HIV and with a current address found nearly 2000 people living with HIV residents were no longer living in DC between 2012 and 2016, with over 80% relocating to surrounding areas [21]. Further, this analysis found differences in relocation by race/ethnicity, gender identity, and mode of transmission, and those who moved out of DC were more likely to be male, black, between the ages of 30 and 39 years, and have a mode of HIV transmission of MSM [21].

Univariate analysis revealed differences in HIV-related health outcomes but after adjusting for demographics, time since HIV diagnosis, and mode of HIV transmission, DC Cohort participants continued to be significantly more engaged in HIV care and have better clinical outcomes compared with noncohort participants. Age at the end of 2017 and mean time since HIV diagnosis most impacted this result, indicating that older age and longer duration of HIV diagnosis were associated with having more recent viral suppression among people living with HIV in DC, which is similar to findings in past research [28-31].

### Limitations

Although this analysis provides insight into whether DC Cohort study participants represent the general HIV population in DC, it was subject to several limitations. First, it was limited to only those who were living in DC at the end of 2016, excluding patients who live outside of the jurisdiction but receive care in DC. In the DC Cohort, approximately 25% of participants are non-DC residents at the time of enrollment and thus were excluded from this analysis. Although these patients were excluded, including them would not have changed the demographic differences between cohort and noncohort participants, as a sensitivity analysis showed that there were still variations by race/ethnicity, gender identity and mode of HIV transmission (Multimedia Appendix 1). Furthermore, lab results from those who live outside of DC are not routinely reported to DCDOH. Second, surveillance-based lab data were used to quantify HIV clinical outcomes, including median CD4 count, HIV stage, receipt of care, and viral suppression. Analyses of these variables were based on lab data reported to DCDOH surveillance databases. If residents were diagnosed in DC but later moved out of the city, lab information may no longer be reported to the local health department, resulting in an underreporting of clinical outcomes. Third, neither ART use nor treatment adherence were measured in this analysis. Although this information would better characterize differences between DC Cohort and noncohort patients, these data are not routinely submitted to DCDOH for all people living with HIV. Further, this analysis does not categorize noncohort participants who were approached and declined to participate or those who have not yet been approached. Although these data would give insight into any self-selection bias that may have occurred among patients, this information is not reported to the health department and therefore not included in this analysis. Moreover, the DC Cohort data provides information that is not routinely disclosed to DCDOH, including information on antiretroviral use, employment, housing status, insurance type, and other non–HIV-related laboratory tests, diagnoses, and treatments. Finally, linkage results have shown that the DC Cohort study provides additional VL lab results to DCDOH that may not have been previously reported [20]. These data were not used as part of this analysis as we relied solely on surveillance data to make the comparisons.

### Conclusion

Although participants from the DC Cohort study may not represent the broader citywide population of people living with HIV, they do provide an important snapshot of HIV care and related clinical outcomes that can assist with understanding the quality of HIV care delivery in a highly impacted urban area. In conducting this analysis, we identified variances between the two groups and intend to use these findings from a practical level to increase the number of patients who are approached at current participating sites to improve the study’s representativeness; at a statistical level, we could consider developing weights to apply to DC Cohort data to increase its generalizability. Despite its limited representativeness in some respects, the DC Cohort study still enhances our ability to describe, monitor, and improve outcomes among large numbers of people living with HIV receiving care in DC. The DC Cohort study provides information on insurance status, clinic visits, ART prescribing, behaviors such as smoking and alcohol use, screening for certain conditions, and other comorbidities (eg, cardiovascular, metabolic) that are not routinely captured in surveillance data yet are useful in contextualizing clinical outcomes among people living with HIV. In addition, the ability to link data between DCDOH and the DC Cohort study is of added value to both researchers and DOH as we aim to address the epidemic. As participants and health care facilities continue to enroll in the DC Cohort study, ongoing assessment of representativeness will be required.

## Appendix

Multimedia Appendix 1 Demographic characteristics of DC Cohort and non-DC cohort participants as of June 2017, including non-DC residents (n=15,273).

## Abbreviations

ART: antiretroviral therapy
DC: District of Columbia
DCDOH: DC Health
EMR: electronic medical record
HAHSTA: HIV/AIDS, Hepatitis, STD, and TB Administration
HBV: hepatitis B virus
HCV: hepatitis C virus
HPTN: HIV Prevention Trials Network
ID: identification
MSM: men who have sex with men
NIAID: National Institute of Allergy and Infectious Diseases
NIH: National Institutes of Health
STI: sexually transmitted infection
TLC-Plus: the Test, Link-to-Care Plus Treat
VL: viral load
