# Supplementary material for: Comparison of Clinical Outcomes of Persons Living With HIV by Enrollment Status in Washington, DC: Evaluation of a Large Longitudinal HIV Cohort Study
Source: JMIR Public Health Surveill. 2020 Apr 15;6(2):e16061. doi: 10.2196/16061 (PMC7191350; doi:10.2196/16061)
Supplement: Multimedia Appendix 1 [file publichealth_v6i2e16061_app1.docx]

**Supplemental Table**. Demographic characteristics of DC Cohort and Non-DC Cohort participants as of June 2017, including Non-DC residents (N=15,273).

|  | **DC Cohort** | **Non-DC Cohort**^a^ | **Total** | **χ^2^** |
| --- | --- | --- | --- | --- |
| **Characteristic** | **N (%)** | **N (%)** | **N** | **p-value** |
|  | 7,502 | 7,771 | 15,273 |  |
| **Gender Identity** |  |  |  |  |
| Male | 5,299 (70.6) | 5,818 (74.9) | 11,117 | <0.0001 |
| Female | 2,110 (28.1) | 1816 (23.4) | 3,926 |  |
| Transgender | 93 (1.2) | 137 (1.5) | 230 |  |
| Race/ethnicity |  |  |  |  |
| White | 955 (12.7) | 1561 (20.1) | 2,516 | <0.0001 |
| Black | 5831 (77.7) | 5,399 (69.5) | 11,230 |  |
| Hispanic | 461 (6.1) | 582 (7.5) | 1043 |  |
| Other^b^ | 255 (3.4) | 229 (3.0) | 484 |  |
| Transmission risk |  |  |  |  |
| MSM | 2981 (39.7) | 3,764 (48.5) | 6,745 | <0.0001 |
| IDU | 977(13.0) | 604 (7.8) | 1,581 |  |
| MSM/IDU | 282(3.8) | 219 (2.8) | 501 |  |
| Heterosexual contact | 2,097 (27.9) | 2,014 (25.9) | 4,111 |  |
| Perinatal | 94 (1.3) | 43 (0.6) | 136 |  |
| Other^c^ | 129 (1.7) | 7 (0.1) | 136 |  |
| Race/ethnicity | 942 (12.6) | 1,121 (14.4) | 2,063 |  |
| **Age as of December 31, 2017** |  |  |  |  |
| Median (IQR) | 50 (18) | 48 (20) |  | 0.7314 |
| **Time since HIV disease diagnosis** |  |  |  |  |
| Mean (SD) | 12.8 (7.1) | 10.7 (7.4) |  |  |
| **Ever STD as of 2011-2016** | 1,296 (17.3) | 1,471(18.9) | 2,767 | 0.0328 |
| **Ever Hepatitis B co-infection (2011-2016)** | 101 (1.3) | 100 (1.3) | 201 | 0.853 |
| **Ever Hepatitis C co-infection (2011-2016)** | 517 (6.9) | 345 (4.4) | 862 | 0.001 |

^a^ Non-DC Cohort participants include persons who have consented and subsequently withdrawn from the study, as well as persons diagnosed with HIV and reported to the DC Department of Health who were alive as of the end of December 2017.

^b^ Other race includes mixed race individuals, Asians, Alaska Natives, American Indians, Native Hawaiian, Pacific Islanders, and unknown race.

^c^ Other mode of transmission includes perinatal transmission, hemophilia, blood transfusion, and occupational exposure (healthcare workers).
